# Pharmacokinetics and Pharmacodynamics (PK/PD) of Corallopyronin A against Methicillin-Resistant *Staphylococcus aureus*

**DOI:** 10.3390/pharmaceutics15010131

**Published:** 2022-12-30

**Authors:** Katharina Rox, Tim Becker, Andrea Schiefer, Miriam Grosse, Alexandra Ehrens, Rolf Jansen, Tilman Aden, Stefan Kehraus, Gabriele M. König, Anna K. Krome, Marc P. Hübner, Karl G. Wagner, Marc Stadler, Kenneth Pfarr, Achim Hoerauf

**Affiliations:** 1 Department of Chemical Biology, Helmholtz Centre for Infection Research (HZI), Inhoffenstrasse 7, 38124 Braunschweig, Germany; 2 German Center for Infection Research (DZIF), Partner-Site Hannover-Braunschweig, 38124 Braunschweig, Germany; 3 Department of Pharmaceutical Technology and Biopharmaceutics, University of Bonn, Gerhard-Domagk-Strasse 3, 53121 Bonn, Germany; 4 German Center for Infection Research (DZIF), Partner-Site Bonn-Cologne, 53127 Bonn, Germany; 5 Institute for Medical Microbiology, Immunology and Parasitology, University Hospital Bonn, Venusberg-Campus 1, 53127 Bonn, Germany; 6 Department of Microbial Drugs, Helmholtz Centre for Infection Research (HZI), Inhoffenstrasse 7, 38124 Braunschweig, Germany; 7 Institute for Pharmaceutical Biology, University of Bonn, Nussallee 6, 53115 Bonn, Germany

**Keywords:** natural product, pharmacokinetics, pharmacodynamics, *Staphylococcus aureus*, PK/PD

## Abstract

Methicillin-resistant *Staphylococcus aureus* (MRSA) is a World Health Organization’s high priority pathogen organism, with an estimated > 100,000 deaths worldwide in 2019. Thus, there is an unmet medical need for novel and resistance-breaking anti-infectives. The natural product Co-rallopyronin A (CorA), currently in preclinical development for filariasis, is efficacious against MRSA *in vitro*. In this study, we evaluated the pharmacokinetics of CorA after dosing in mice. Furthermore, we determined compound concentrations in target compartments, such as lung, kidney and thigh tissue, using LC-MS/MS. Based on the pharmacokinetic results, we evaluated the pharmacodynamic profile of CorA using the standard neutropenic thigh and lung infection models. We demonstrate that CorA is effective in both standard pharmacodynamic models. In addition to reaching effective levels in the lung and muscle, CorA was detected at high levels in the thigh bone. The data presented herein encourage the further exploration of the additional CorA indications treatment of MRSA- and methicillin-sensitive *S. aureus*- (MSSA) related infections.

## 1. Introduction

Antimicrobial resistance is a persistent threat to human health with several million individuals affected around the globe [1]. The World Health Organization (WHO) has set up a priority list for pathogens for which new treatment options are desperately needed [2,3]. Among these pathogens is methicillin-resistant *Staphylococcus aureus* (MRSA), which is estimated to be responsible for more than 100,000 deaths in 2019 alone [1]. These facts highlight the importance of finding novel antibiotics and innovative therapy concepts as the development pipeline runs dry [4].

Corallopyronin A (CorA) was discovered in the mid-1980s [5], but only during the recent ten years has it started to gain more attention [6]. CorA targets the RNA-polymerase, but binds to the ‘switch-region’, a different binding site compared to rifampicin [7,8,9]. As a result, CorA has broad-spectrum antibacterial activity and, at the same time, does not select for cross-resistance with rifampicin [10]. Thus, CorA shows activity against a plethora of pathogens, in particular intracellular such as gonococci [11], *Chlamydia* spp. [12,13], *Orientia tsutsugamushi* and *Rickettsia typhi* [14], as well as *Staphylococcus aureus* [15,16] and the filarial endo-symbiont *Wolbachia*—the activity against the latter one is the basis of treatment of the neglected tropical diseases lymphatic filariasis and onchocerciasis [17,18,19,20]. Due to an optimized biosynthetic production process in a heterologous host [21], it has become feasible to upscale production to amounts of CorA needed for (pre-)clinical development. Recently, the ADME as well as some early pharmacokinetic (PK) properties and the preliminary safety profile of CorA have been reported and provide confidence for development towards the clinics in the indication for filariasis [22]. In a recent study, it was furthermore shown that CorA had a lower frequency of resistance (FoR) for *S. aureus* than rifampicin [16]. Initially, the FoR of CorA against *S. aureus* was estimated to be higher [15], but that study did not consider the chemical instability of unformulated CorA resulting in an overestimation of the FoR [16].

Taking into account the lower FoR, we aimed to investigate if CorA was also active *in vivo* against MRSA to elucidate if CorA is suitable for development against MRSA infections. Thus, this study provides an in-depth pharmacokinetic (PK) characterization of CorA and demonstrates that it is effective in both standard pharmacodynamic (PD) models.

## 2. Materials and Methods

### 2.1. Chemicals

CorA with a purity > 91% was produced using the heterologous producer strain *My-xococcus xanthus* carrying the CorA biosynthetic gene cluster and was purified as reported previously [17,21,23]. Levofloxacin was purchased from Sigma-Aldrich (Taufkirchen, Germany). Tralieve^®^ (Tramadol 50 mg/mL, Dechra veterinary products, Aulendorf, Germany) was diluted 1:8 in 0.9% NaCl-solution.

### 2.2. Pharmacokinetic Studies

For pharmacokinetic (PK) experiments, 6–8-week-old outbred female CD-1 mice (Charles River, Germany) were used. CorA was dissolved in PEG400/phosphate-buffered saline (PBS) (80:20 (*v*/*v*)). CorA was administered once daily at 60 mg/kg intraperitoneally (IP), 100 mg/kg IP, 100 mg/kg subcutaneously (SC) and 100 mg/kg perorally (PO). Moreover, CorA was administered at 30 mg/kg IP twice a day (BID) (administration time points t = 0 and 6 h after first dose). Animals (*n* = 3 per time point) receiving either 60 mg/kg IP or 100 mg/kg IP were euthanized at 10, 15, 30, 60 and 180 min after administration. Animals (*n* = 3 per time point) receiving 30 mg/kg IP BID were euthanized at 0.167, 0.25, 0.5, 1, 3, 5.75, 6.167, 6.25, 6.5, 7 and 9 h after first administration. Animals (*n* = 3 per time point) receiving 100 mg/kg SC or 100 mg/kg PO were euthanized at 0.25, 1, 2, 4 and 8 h after administration. At every time point blood was removed from the heart, collected into tubes coated with 0.5 M ethylenediaminetetraacetic acid (EDTA), immediately spun down at 15,870× *g* for 10 min at 4 °C and the resultant plasma transferred into a new tube. Furthermore, at every time point heart, lung, brain, liver, spleen, kidneys, and thigh muscles were removed and frozen in liquid nitrogen. For animals receiving 100 mg/kg PO also rectum (without feces), pharynx, skin, vaginal tract, subcutaneous fat tissue, visceral fat tissue and tibiae were removed and frozen in liquid nitrogen. Before removing the vaginal tract, a vaginal flush was performed.

### 2.3. Bioanalytics of PK and PD Studies

#### 2.3.1. Bioanalytics of Plasma Samples

CorA plasma concentrations were quantified by HPLC-DAD with an Alliance e2695 separation module and a 2998 PDA detector (Waters, Eschborn, Germany). For the analysis, blood samples were centrifuged for 10 min at 4 °C and 3220× *g*. The generated plasma (20 µL) was mixed 1:3 with ice-cold acetonitrile, vortexed for 10 s and centrifuged for 25 min at 4 °C and 11,000× *g*. The supernatant was transferred into a HPLC vial and a sample volume of 5 µL was injected and quantified at a wavelength of 300 nm. A Waters XBridge^®^ Shield RP18 column (3.5 μm, 2.1 × 100 mm, 130 A) was used at 30 °C. Two solvent gradients comprising mobile phase A (acetonitrile/water 5/95 with 5 mM ammonium acetate and 40 μL acetic acid per liter) and mobile phase B (acetonitrile/water 95/5 with 5 mM ammonium acetate and 40 μL acetic acid per liter) with a gradient from 70%A/30%B to 20%A/80%B, stepwise within 30 min and a flow rate of 0.3 mL/min were used. Data were analyzed by Empower 3 software and quantified using an external reference standard. No major matrix effects were seen when mixing ACN containing CorA with plasma [22].

#### 2.3.2. Bioanalytics of Organ Samples (PK and PD Samples)

Organ samples were thawed and homogenized using a Polytron tissue homogenizer after addition of 3 mL isotonic sodium chloride solution. First, a calibration curve was prepared by spiking different concentrations of CorA into the respective matrix from CD-1 mice. Caffeine was used as an internal standard. In addition, quality control samples (QCs) were prepared for CorA for every matrix. For PD samples, calibration curve and QCs were prepared for levofloxacin using the same protocol. The following extraction procedure was used: 50 µL of a sample (calibration sample, QCs, PK or PD sample) was extracted with 20 µL of acetic acid in water (10/90 (*v*/*v*)) and 30 µL acetonitrile for 5 min at 800 rpm on an Eppendorf MixMate^®^ vortex mixer. Then, samples were spun down for 20 min at 4 °C and 4000 rpm and supernatants were transferred to new Greiner 96-well V-bottom plates. Samples were analyzed using an Agilent 1290 Infinity II HPLC system (Waldbronn, Germany) coupled to an AB Sciex QTrap6500plus mass spectrometer (Darmstadt, Germany). LC conditions were as follows: column: Agilent Zorbax Exlipse Plus C18, 50 × 2.1 mm, 1.8 µm; temperature: 30 °C; injection volume: 5 µL per sample; flow rate: 800 µL/min. Samples were run under buffered conditions: A: 95% water + 5% acetonitrile + 5 mM ammonium acetate + 40 µL/L acetic acid; B: 95% acetonitrile + 5% water + 5 mM ammonium acetate + 40 µL/L acetic acid. The gradient was as follows: 99% A at 0 min, 99% A until 0.1 min, 99–0% A from 0.1 min to 4.0 min, 0% A until 4.5 min. Mass transitions for controls and compounds are depicted in Table S1. Peaks of PK samples were quantified using the calibration curve. The accuracy of the calibration curve was determined using QCs independently prepared on different days. PK parameters were determined using a non-compartmental analysis with PKSolver [24].

### 2.4. Preparation of the Inoculum for Infection with S. aureus ATCC 33591

Inocula were prepared as described previously with the modification that isotonic sodium chloride solution was used instead of PBS [25]. For the neutropenic thigh and the neutropenic lung infection model the *S. aureus* strain ATCC 33591 (MRSA-strain) was used. For the neutropenic lung infection model an inoculum of 2 × 10^9^ cfu/mL was used. For the neutropenic thigh infection model an inoculum of 7 × 10^5^ cfu/mL was used.

### 2.5. Neutropenic Thigh Infection Model with MRSA

The model was conducted as described previously [25]. In brief, the following groups were used: (1) inoculum control group with terminal endpoint at 2 h post infection to determine baseline inoculum *in vivo*; (2) vehicle-treated group receiving 20 mg/kg SC tramadol and vehicle (PEG400/PBS (80:20 (*v*/*v*)); (3) levofloxacin group 5 mg/kg IP three times a day (TID) (t = 2, 6 and 10 h post infection); (4) CorA 60 mg/kg IP TID (t = 2, 6 and 10 h post infection) and (5) CorA 100 mg/kg IP BID (t = 2 and 6 h post infection). The endpoint for groups no. 2–5 was 24 h post infection. A solution of CorA in PEG400/PBS (80:20 (*v*/*v*)) was used as vehicle.

### 2.6. Neutropenic Lung Infection Model with MRSA

Female, 8-week-old CD-1 mice (Charles River, Sulzfeld, Germany) were used (*n* = 6 animals per group). Animals were rendered neutropenic by administration of 150 mg/kg and 100 mg/kg cyclophosphamide IP on days -4 and -1, respectively. On the day of infection (day 0), mice received 15 µL of the inoculum administered via an Aeroneb^®^ nebulizer device (Kent Scientific, Torrington, CT, USA) under anesthesia (100 mg/kg ketamine and 10 mg/kg xylazine). Mice were treated with (I) vehicle IP (PEG400/PBS), (II) with levofloxacin at 100 mg/kg IP (t = 2 h post infection, positive control group), (III) CorA at 60 mg/kg IP TID (t = 2, 5 and 9 h post infection) or (IV) CorA at 60 mg/kg IP (t = 2 h post infection) and 100 mg/kg SC BID (t = 5 and 9 h post infection).

Twenty-four hours after infection, mice were euthanized, blood was removed from the heart and lungs were aseptically removed. Whole blood was collected into Eppendorf (Hamburg, Germany) tubes coated with 0.5 M EDTA, immediately spun down at 13,000 rpm for 10 min at 4 °C, the plasma transferred into a new Eppendorf tube and stored at −80 °C until analysis. Organs were homogenized in isotonic sodium chloride solution and plated onto blood agar plates in duplicates in serial dilutions and incubated at 37 °C for 24 h.

### 2.7. Animals

The animal studies were conducted in accordance with the recommendations of the European Community (Directive 86/609/EEC, 24 November 1986; EU Directive 2010/63/EU). All animal procedures were performed in strict accordance with the German regulations of the Society for Laboratory Animal Science (GV- SOLAS) and the European Health Law of the Federation of Laboratory Animal Science Associations (FELASA). Animals were excluded from further analysis if euthanasia was necessary according to the humane endpoints established by the ethical board. All efficacy experiments were approved by the ethical board of the Niedersächsisches Landesamt für Verbraucherschutz und Lebensmittelsicherheit, Oldenburg, Germany. All PK studies were approved by the ethical board of the Landesamt für Natur, Umwelt und Verbraucherschutz Nordrhein-Westfalen, Recklinghausen, Germany (#81-02.04.2020.A244). Animals were kept in individually ventilated cages with a 10 h/14 h (HZI) or 12 h/12 h (University Hospital Bonn) dark/light cycle and had access to food and water *ad libitum*. The study is reported in accordance with the ARRIVE guidelines.

### 2.8. Statistical Analysis

Testing for statistical significance was performed using a Kolmogorov–Smirnov-test (unpaired, two-tailed) using GraphPad Prism 9.3.1 software. Results were considered statistically significant when *p*-values were ≤ 0.05 (95% confidence interval).

## 3. Results

### 3.1. Pharmacokinetic Evaluation of CorA

**Figure 1:**
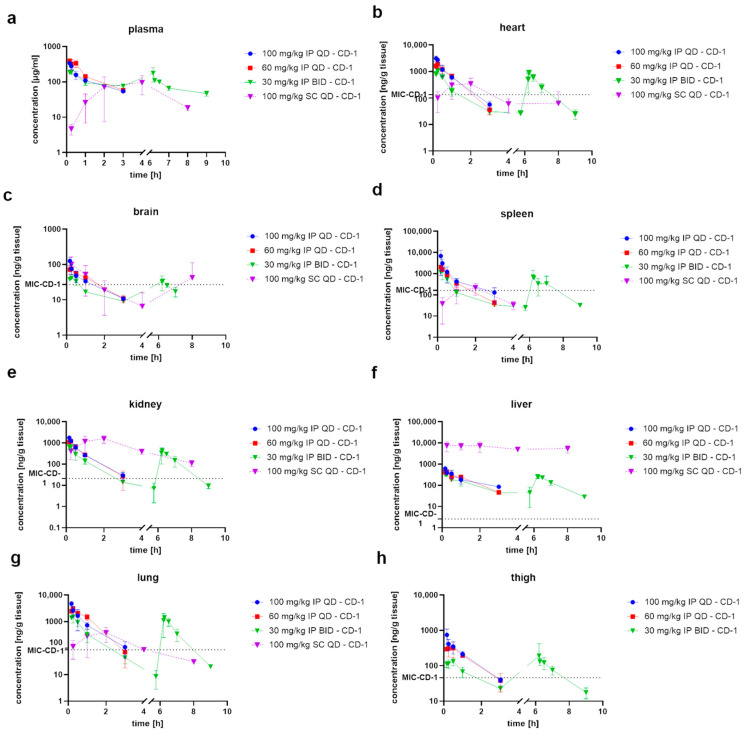
Pharmacokinetic evaluation and biodistribution of CorA after administration of 30, 60 and 100 mg/kg IP as well as after 100 mg/kg SC in CD-1. Concentrations in plasma (**a**), heart (**b**), brain (**c**), spleen (**d**), kidney (**e**), liver (**f**), lung (**g**) and thigh (**h**) are displayed as mean and standard deviation (*n* = 3 per time point).

We first conducted PK studies to investigate the plasma levels and distribution of CorA to the different organs. The PK studies were conducted in CD-1 mice using 30 mg/kg BID, 60 mg/kg once a day (QD) and 100 mg/kg QD IP dosing of CorA. Additionally, CorA was dosed at 100 mg/kg QD SC and PO. For the routes using IP administration, plasma concentrations peaked directly after injection, whereas subcutaneous administration showed a delayed T_max_ and a lower C_max_ compared to the 100 mg/kg IP dose (Figure 1a). BID dosing of 30 mg/kg IP resulted in similar plasma levels as CorA seemed to be completely cleared from plasma when the second dose was administered 6 h after the first dose. This was reflected in similar C_max_ and T_max_ values as well as a similar exposure reflected by the AUC_0-t_ (Table 1). Although higher IP doses of CorA resulted in increased C_max_ values, exposures did not increase in a linear manner to dose for all three dose levels tested: no increase in exposure or C_max_ was observed in plasma when dose was increased from 60 mg/kg to 100 mg/kg IP (Table 1). Although exposure after 100 mg/kg IP dosing of CorA was only slightly lower compared to 100 mg/kg SC dosing, CorA was still found at concentrations of > 10 µg/mL after SC dosing in plasma after 8 h, which was not the case for the same dose using the IP route (Figure 1a, Table 1). Dosing of 100 mg/kg PO resulted in an early T_max_ of ~0.5 h, which varied slightly among individual animals, with a C_max_ 3- to 4-fold lower compared to the same dose administered IP (Table S2). Furthermore, PO administration gave a 3- to 4-fold lower exposure in plasma compared to the same dose administered IP or SC. Nevertheless, CorA was found in plasma up to 8 h after PO dosing (Figure S1a).

We next investigated the biodistribution in heart, brain, liver, spleen, lung, kidney and thigh tissues. For heart tissue, C_max_ increased nearly linear to dose. Whereas exposure in heart tissue at 60 mg/kg IP was linear to the 30 mg/kg IP dose, exposure at 100 mg/kg IP did not result in a linear increase indicating a ceiling effect. The 100 mg/kg SC dose resulted in a delayed T_max_ and a lower C_max_ compared to the 100 mg/kg IP dose. Similar correlations with respect to dose-dependency for C_max_-values after IP dosing were observed for brain, spleen, liver, kidney, lung and thigh tissue (Table 2). For all doses administered via the IP route CorA was only found 3 to 4 h post administration in most of the tissues assessed, whereas SC dosing resulted in a more prolonged profile (Figure 1b–h).

In general, the organ levels after administration of three different doses IP or via 100 mg/kg SC exceeded the *in vitro* minimal inhibitory concentration (MIC) (0.125 µg/mL) [16] for about one hour (Figure 1c). Similar exposures in brain tissue were determined for all three doses administered via the IP route. However, exposure of CorA in brain tissue was much lower compared to other tissues suggesting that CorA did not cross the blood-brain-barrier. Surprisingly, CorA exhibited high concentrations in kidney as well as in liver tissue after SC administration, exceeding the *in vitro* MIC of CorA against MRSA for more than 9 h. That was not observed after IP administration. In lung tissue, all three doses administered IP resulted in CorA levels exceeding the *in vitro* MIC for around 3 h, whereas administration of 100 mg/kg SC led to CorA levels above the MIC for ~4 h (Figure 1g). In thigh tissue, no CorA levels were detected after 100 mg/kg SC dosing. The 60 mg/kg and the 100 mg/kg IP dose achieved CorA levels lasting around 3 h above the MIC. The 30 mg/kg IP dose only showed CorA levels exceeding the MIC for about one hour (Figure 1h). For all tissues assessed, the second dose of 30 mg/kg IP resulted in similar CorA levels as the first dose (Figure 1b–h). Although SC administration resulted in much lower C_max_ levels compared to IP, it showed high exposure, especially in liver, kidney, heart and lung tissues.

Next, we examined biodistribution after PO dosing. Concentrations peaked early, after 0.25 to 0.5 h, in the investigated tissues, i.e., thigh, heart, kidney, spleen, lung, liver, subcutaneous fat, visceral fat, rectum, pharynx, vaginal tract, skin and tibia, but showed more sustained levels and a lower clearance compared to the IP route (Figure S1b,c). However, CorA administered PO was not found after vaginal flush. Finally, PK studies with the three different doses administered IP and the 100 mg/kg dose administered SC and PO showed that, apart from brain, CorA distributes to all tissues investigated. Moreover, it showed that especially IP dosing resulted in high initial peak concentrations in tissue which were above the MIC for 2–3 h. By contrast, SC dosing led to a delayed peak concentration of CorA and longer-lasting concentrations and higher exposures in tissue, although concentrations were much lower compared to IP, except in liver and kidney tissue.

### 3.2. CorA Is Effective in the Neutropenic Thigh Infection Model

We then embarked on *in vivo* efficacy studies using two standard pharmacodynamic models, the neutropenic thigh and the neutropenic lung infection model. First, we assessed CorA using different doses and dosing regimens in the neutropenic thigh infection model with a methicillin-resistant *S. aureus* (MRSA) strain. Two different dosing groups of CorA were used: CorA was administered at 60 mg/kg IP TID and at 100 mg/kg IP BID. At this stage and with the present formulation, we did not deploy the PO route for the *in vivo* efficacy studies as plasma levels were much lower compared to the other routes investigated. Moreover, after SC administration no compound was detected in thigh. Levofloxacin was used as a positive control. After 24 h, the bacterial burden was determined in the primary infection site, thigh, and in lung tissue, affected by secondary (hematogenous) bacterial seeding. CorA dosed at 60 mg/kg IP TID did not show any effect, neither in thigh nor in lung tissue. For both infection sites, the bacterial burden was similar to the vehicle-treated group (Figure S2a,b). Levofloxacin resulted in a significant reduction of bacterial burden in thigh tissue, in the same range as the inoculum control group (Figure S2a,c). In lung tissue, levofloxacin also reduced bacterial burden compared to the vehicle-treated group by approximately 2 log_10_-units (Figure S2b,d). CorA dosed at 100 mg/kg IP BID led to a significant reduction in bacterial burden in thigh, similar to levofloxacin (Figure S2a,c). Next, we assessed terminal compound levels in blood, kidney, liver, heart, lung and thigh. Levofloxacin was only detected at low levels in blood and was not detected in any of the investigated tissues terminally (Figure S3a). CorA dosed at 60 mg/kg IP TID and 100 mg/kg IP BID exhibited similar terminal blood levels (Figure S3a). In liver, the highest levels were detected for CorA at 60 mg/kg IP TID, whereas CorA at 100 mg/kg IP BID had more than 5-fold lower levels (Figure S3b). In kidney and heart tissue, CorA at 60 mg/kg IP TID and at 100 mg/kg IP BID exhibited nearly equal terminal concentrations (Figure S3c,d). In lung tissue, CorA was not detected in the 60 mg/kg IP TID group, but detected at low levels in the 100 mg/kg IP BID group (Figure S3e). In thigh, CorA at 60 mg/kg IP BID showed more than 10-fold lower concentrations than in the group at 100 mg/kg IP BID (Figure S3f). Finally, terminal significant CorA concentrations were only detected in thigh and blood in the 100 mg/kg IP CorA group.

In summary, the high dose group with 100 mg/kg IP CorA BID resulted in a significant reduction in bacterial burden in thigh tissue similar to levofloxacin. By contrast, the 60 mg/kg IP TID dose of CorA failed to show an effect. Although, the total daily dose was nearly similar (180 mg/kg/day vs. 200 mg/kg/day), and thus the total exposures, the same effect was not observed with respect to reduction of bacterial burden in thigh.

### 3.3. CorA Reduces Bacterial Burden in the Neutropenic Lung Infection Model

**Figure 2:**
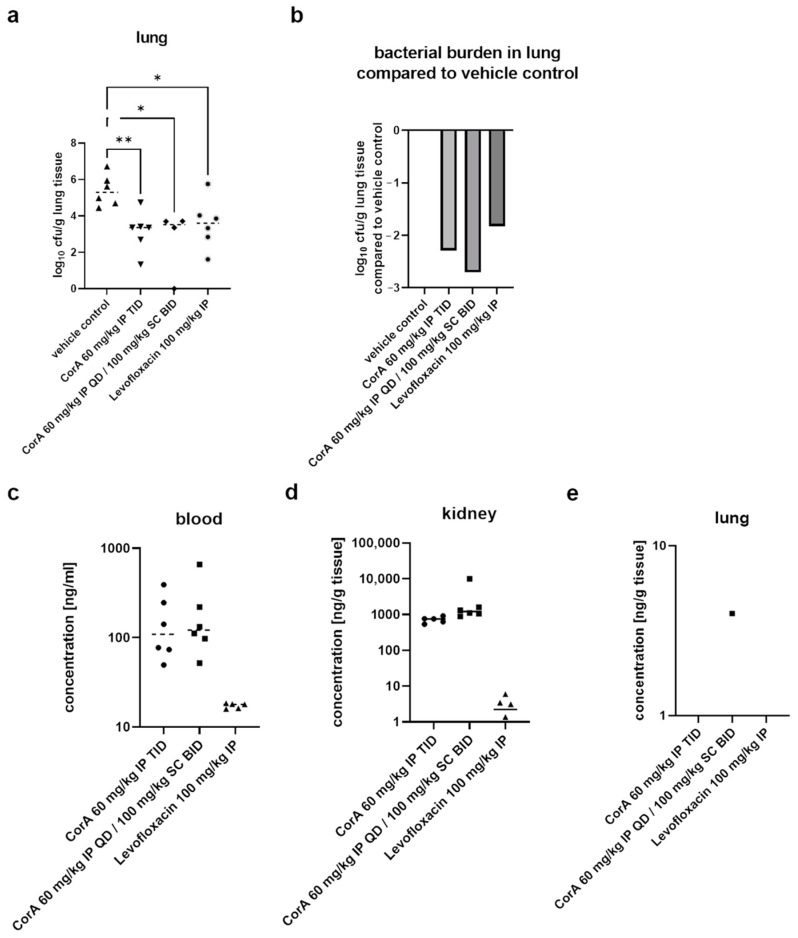
CorA shows efficacy in the neutropenic lung infection model with MRSA. Bacterial burden expressed as log_10_ cfu/g lung tissue is shown for vehicle control group, CorA 60 mg/kg IP TID, CorA 60 mg/kg IP QD + 100 mg/kg SC BID and Levofloxacin 100 mg/kg IP. (**a**). Bacterial burden reduction compared to vehicle control group is displayed for CorA 60 mg/kg IP TID, CorA 60 mg/kg IP QD + 100 mg/kg SC BID and Levofloxacin 100 mg/kg IP (**b**). Compound concentrations in blood (**c**), kidney (**d**) and lung (**e**) are shown for CorA 60 mg/kg IP TID, CorA 60 mg/kg IP QD + 100 mg/kg SC BID and Levofloxacin 100 mg/kg IP. *: *p* < 0.05, **: *p* < 0.01 using a Kruskal–Wallis test.

In a second step, we evaluated CorA in a neutropenic lung infection model with MRSA. Based on the results of the neutropenic thigh infection model, we modified the dosing groups. In the first group we dosed 60 mg/kg IP TID exhibiting high peak concentrations in lung tissue and plasma in a shorter interval to prevent concentrations dropping below MIC before administration of the next dose. In the second group we dosed 60 mg/kg IP followed by two 100 mg/kg SC doses. With this dosing regimen a high initial peak concentration was established and then maintained over MIC with the two subsequent SC administrations. The bacterial burden was only assessed in lung tissue 24 h post infection. Levofloxacin was used as positive control and showed a reduction in bacterial burden by slightly more than 2 log_10_-units. Both CorA groups tested showed a similar reduction in bacterial burden as levofloxacin (Figure 2a,b). When assessing terminal compound levels in blood, kidney and lung, we found that both CorA groups had similar terminal compound levels in blood and kidney tissue whereas levofloxacin showed much lower levels (Figure 2c,d). Finally, no compound was detected terminally in lung tissue apart from one single animal in the CorA group with 60 mg/kg IP + 100 mg/kg SC BID. This suggests that initial high compound concentrations in lung tissue must have been responsible for the effect, as literally no compound was detected terminally (Figure 2e).

In summary, we demonstrated that CorA is effective in both standard pharmacodynamic models as it led to a significant reduction in bacterial burden in the target organs similar to levofloxacin.

## 4. Discussion

CorA is currently advancing through preclinical development for the indication lymphatic filariasis and onchocerciasis [22]. As the regulatory pathways are already undertaken for these indications, it is worth exploring other indications, as no additional safety pharmacology may be needed. This helps to accelerate the process for anti-infective drug development in indications for which antibiotics are desperately needed [4].

### 4.1. Biodistribution Studies Form the Basis for Design of Efficacy Studies

In this study, we investigated the potential of CorA to treat staphylococcal infections. In a first step, we assessed the biodistribution and characterized levels of CorA in different target organs at different doses and three distinct routes of administration, SC, PO and IP. Although CorA had already been tested for its PK properties *in vivo*, this study was the first one to systematically determine the biodistribution after SC, PO and IP administration [17,20]. For the 30 mg/kg and 60 mg/kg doses administered IP, dose-linearity was observed in plasma, whereas a ceiling effect was observed after administration of 100 mg/kg IP. By contrast, dose-linearity was seen for all three dose levels for the majority of organs assessed in this study. This suggests a more rapid clearance from plasma at the 100 mg/kg IP dose. It is interesting that SC administration did not support distribution towards thigh, whereas IP administration of the same dose resulted in peak concentrations in thigh more than 3–5× above the *in vitro* determined MIC of 0.125 µg/mL against MRSA [16]. This highlights the importance to investigate biodistribution ahead of efficacy experiments. At first sight, it is astonishing that CorA was found in brain, although to only a low extent. This might be explained by its high lipophilicity as lipophilic compounds can cross the blood-brain-barrier to a low extent via passive diffusion if plasma concentrations are sufficiently high [22,26]. Thus, compound concentrations measured in brain might be only due to high plasma concentrations and not a result of crossing the blood-brain-barrier. CorA is a lipophilic compound which is mainly cleared by the liver and then excreted by the kidneys, similar to other lipophilic drugs, and therefore, following typical ADME rules [27,28]. In contrast to thigh, SC administration gave much higher levels in liver and kidney compared to the different doses administered IP, suggesting a different biodistribution after SC administration that resulted in a reservoir, continuously liberating CorA and leading to much higher levels in kidney and liver. Finally, SC administration also resulted in good exposures in heart tissue suggesting investigating effectiveness in models of staphylococcal endocarditis for future research.

### 4.2. CorA Shows Favorable Biodistribution after PO Administration Paving the Way for Additional Applications

In addition to the investigation of biodistribution towards tissues, organ concentrations were assessed in further organs after PO administration. Although in this study only low bioavailability was seen after PO administration compared to the IP and SC routes, recent studies have shown and predicted good bioavailability using a povidone-based amorphous solid dispersion method, paving the way to deploy this formulation for additional efficacy studies [23,29]. Whereas high rectum and pharynx concentrations of CorA might be attributed to the administration route, it was surprising to find CorA in the vaginal tract tissue as well as in tibia. The biodistribution towards these tissues might pave the way for further indications, such as treatment of staphylococcal osteomyelitis, or treatment of gonococcal diseases as CorA is also potent against gonococci [11]. Especially for staphylococcal osteomyelitis, it would be beneficial to achieve high organ concentrations after PO administration as long-term treatment is usually needed in this indication [30]. However, further investigations have to prove efficacy in that indication first. Nevertheless, it should also be kept in mind that tissue concentrations are determined from homogenates, neglecting the fact that tissues are composed of distinct compartments and, therefore, look at the tissue concentration as an average of the whole [31,32]. This has to be considered when evaluating PK/PD effects only based on tissue concentrations. Therefore, it is advisable to always look at measured tissue concentrations in context with plasma concentrations. Although plasma concentrations are still one of the best surrogate parameters to determine the PK/PD drivers, they might not accurately reflect the distribution to specific target compartments [33].

### 4.3. In Vivo Proof of Concept of CorA as a Basis for Further Development

PK/PD drivers have been established some time for antibiotic development [34]. These parameters help to understand which PK results in a PD effect that also translates to humans [34,35,36]. We showed that CorA was effective in both standard models for PD evaluation of antibiotics. Biodistribution and tissue concentrations help to understand possible effects and PD behavior and contribute to optimal design of efficacy trials. In the neutropenic thigh infection model similar exposures at the target tissue and in plasma did not result in the same effect. In the neutropenic lung infection model, the group with 60 mg/kg CorA IP followed by 100 mg/kg SC BID showed a slightly better effect, presumably as a result of longer time over MIC in plasma and in the target tissue, the lung. However, further investigations, using for example dose fraction studies [35], are needed to validate this first estimation. Both standard PD models together helped to gain first insights into PK/PD correlations.

Moreover, information about measured tissue and plasma concentrations as well as the PD effect fed into *in silico* models might help to understand PK/PD drivers in a better way. As tissues can be represented with their compartmental (sub-)structures in *in silico* models, this allows to calculate the PD effect in specific target tissues [37,38]. A first rough estimation of human equivalent doses using the concept of Reagan-Shaw and colleagues [39] based on the efficacy data from this study shows that 100 mg/kg would be equivalent to 8.1 mg/kg human dose, whereas a dose of 60 mg/kg has a human equivalent dose of 4.9 mg/kg. This underlines the potential for further development of CorA. The clear advantage of CorA is that it is not cross-resistant to rifampicin [10,16] and that efforts to assure a good bioavailability have already been undertaken [23]. A good peroral bioavailability of CorA is achievable in clinical practice for the envisaged indications onchocerciasis and lymphatic filariasis [18,20]. Moreover, it is an additional asset for treatment of staphylococcal infections as an intravenous, immediate treatment as well as an oral-step-down therapy might be possible. CorA is Ongoing research into CorA activity against staphylococcal disesases, e.g., osteomyelitis, will provide the data needed to support its clinical use for this secondary indication.

## 5. Conclusions

In summary, we have demonstrated that CorA, currently in preclinical development for the indication lymphatic filariasis and onchocerciasis, distributes well to target tissues and is effective in thigh and lung standard PD models. These investigations give first ideas about the PK/PD driver, which will be subject to further investigation. Finally, this study underlines that CorA can be an effective treatment option against staphylococcal infections and that this indication should be pursued further for preclinical development. Additionally, the biodistribution results after PO administration suggest also exploring other indications, such as staphylococcal osteomyelitis or gonococcal diseases.

## Figures and Tables

**Table 1:** PK parameters in plasma after administration of different doses of CorA using two distinct routes (mean ± SD).

	100 mg/kg IP	60 mg/kg IP	30 mg/kg IP	100 mg/kg SC
t1/2 [h]	1.87 ± 0.8	1.10 ± 0.2	2.33 ± 0.3	
T_max_ [h]	0.19 ± 0.0	0.19 ± 0.0	0.19 ± 0.0	3.33 ± 1.2
C_max_ [µg/mL]	349.40 ± 12.4	416.03 ± 39.11	194.13 ± 15.6	107.1 ± 59.2
AUC**_0-t_** [µg/Ml × h]	339.53 ± 28.4	463.87 ± 18.8	303.88 ± 21.9	459.32 ± 238.2
MRT [h]	2.51 ± 1.0	1.55 ± 0.2	3.61 ± 0.6	
Vz/F_obs [l/kg]	0.54 ± 0.2	0.17 ± 0.0	0.18 ± 0.0	
Cl/F_obs [ml/min/kg]	3.43 ± 0.4	1.80 ± 0.0	0.89 ± 0.0	

t1/2: plasma half-life; T_max_: time point at which maximal concentration is reached; C_max_: maximal concentration; AUC_0-t_: Area under the curve from time point 0 until t; MRT: mean residence time; Vz/F_obs: fractionated observed volume of distribution; Cl/F_obs: observed fractionated plasma clearance; IP: intraperitoneally; SC: subcutaneously.

**Table 2:** Maximal concentrations and exposures in tissues after administration of different doses of CorA using two distinct routes (mean ± SD).

	C_max_ [µg/g]				AUC [µg/g × h]			
	IP			SC	IP			SC
Organ	100 mg/kg	60 mg/kg	30 mg/kg	100 mg/kg	100 mg/kg	60 mg/kg	30 mg/kg	100 mg/kg
Liver	0.61	0.43	0.31	2.3	0.59	0.57	0.40	12.44
Heart	3.1	1.8	1.1	1.48	2.10	1.83	1.08	3.53
Spleen	4.9	2.1	1.4	0.6	2.55	1.34	0.77	1.4
Brain	0.1	0.07	0.04	0.15	0.10	0.10	0.05	0.24
Lung	4.8	3.1	1.5	1.27	2.67	3.56	1.2	3.85
Kidney	1.8	1.2	0.7	1.76	1.01	0.95	0.49	5.61
Thigh	0.7	0.3	0.1	-	0.60	0.49	0.19	-

C_max_: maximal concentration; AUC: area under the curve; IP: intraperitoneally; SC: subcutaneously.

## Data Availability

The data presented in this study are available within the manuscript and the supplemental information.

## Supplementary Materials

The following supporting information can be downloaded at: https://www.mdpi.com/article/10.3390/pharmaceutics15010131/s1, Figure S1: Pharmacokinetic evaluation and biodistribution of CorA after administration of 100 mg/kg PO; Figure S2: CorA shows efficacy in the neutropenic thigh infection model with MRSA; Figure S3: Bioanalytical evaluation of CorA and levofloxacin in the neutropenic thigh infection model; Table S1: Mass transitions for quantification of CorA and levofloxacin using caffeine as internal standard; Table S2: PK parameters in plasma after administration of 100 mg/kg PO.
