# Supplementary material for: Pharmacokinetics and Pharmacodynamics (PK/PD) of Corallopyronin A against Methicillin-Resistant Staphylococcus aureus
Source: Pharmaceutics. 2022 Dec 30;15(1):131. doi: 10.3390/pharmaceutics15010131 (PMC9860980; doi:10.3390/pharmaceutics15010131)
Supplement: Supplementary file 1 [file pharmaceutics-15-00131-s001.zip › pharmaceutics-2080277-supplementary.pdf]

pharmaceutics

Supplementary Materials: Pharmacokinetics and Pharmacodynamics (PK/PD) of Corallopyronin A against Methicillin-Resistant *Staphylococcus aureus*

Katharina Rox, Tim Becker, Andrea Schiefer, Miriam Grosse, Alexandra Ehrens, Rolf Jansen, Tilman Aden, Stefan Kehraus, Gabriele M. König, Anna K. Krome, Marc P. Hübner, Karl G. Wagner, Marc Stadler, Kenneth Pfarr and Achim Hoerauf

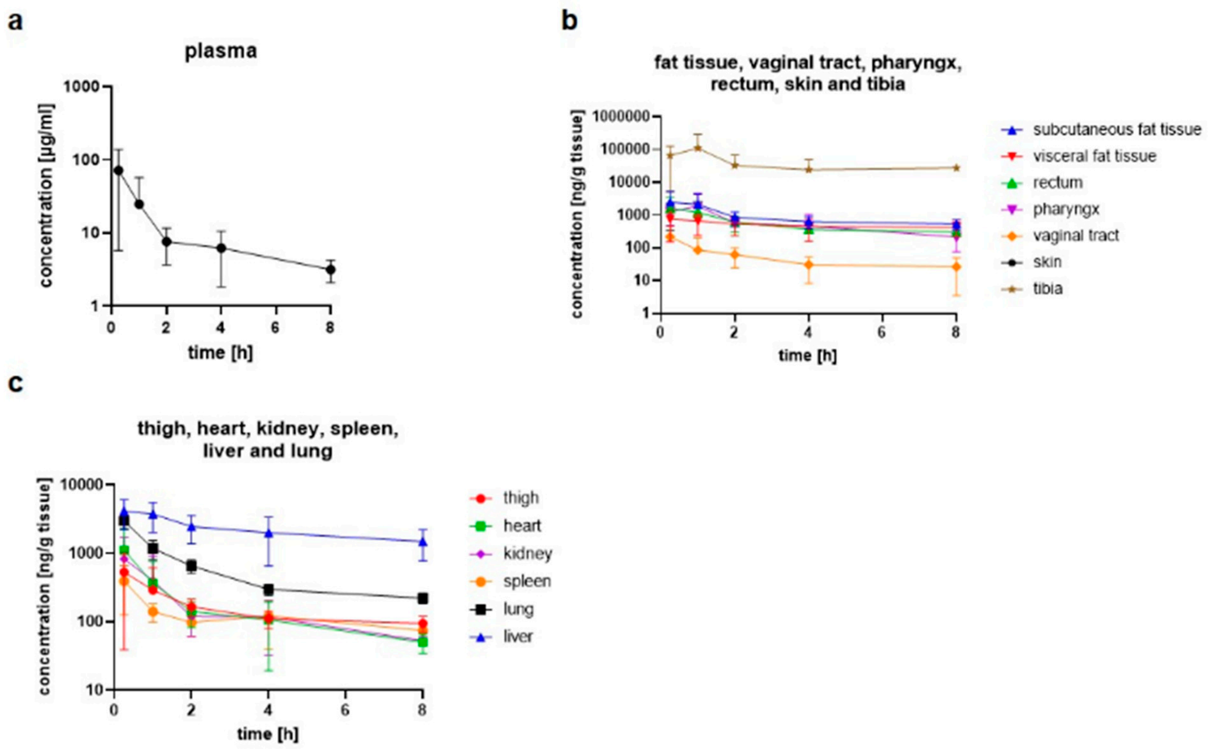

Figure S1. Pharmacokinetic evaluation and biodistribution of CorA after administration of 100 mg/kg PO. Concentrations in plasma (a), subcutaneous fat tissue, visceral fat tissue, rectum pharyngx, vaginal tract, skin and tibia (b) as well as in thigh, heart, kidney, spleen, lung and liver (c) are displayed as mean and standard deviation (*n* = 3 per time point).

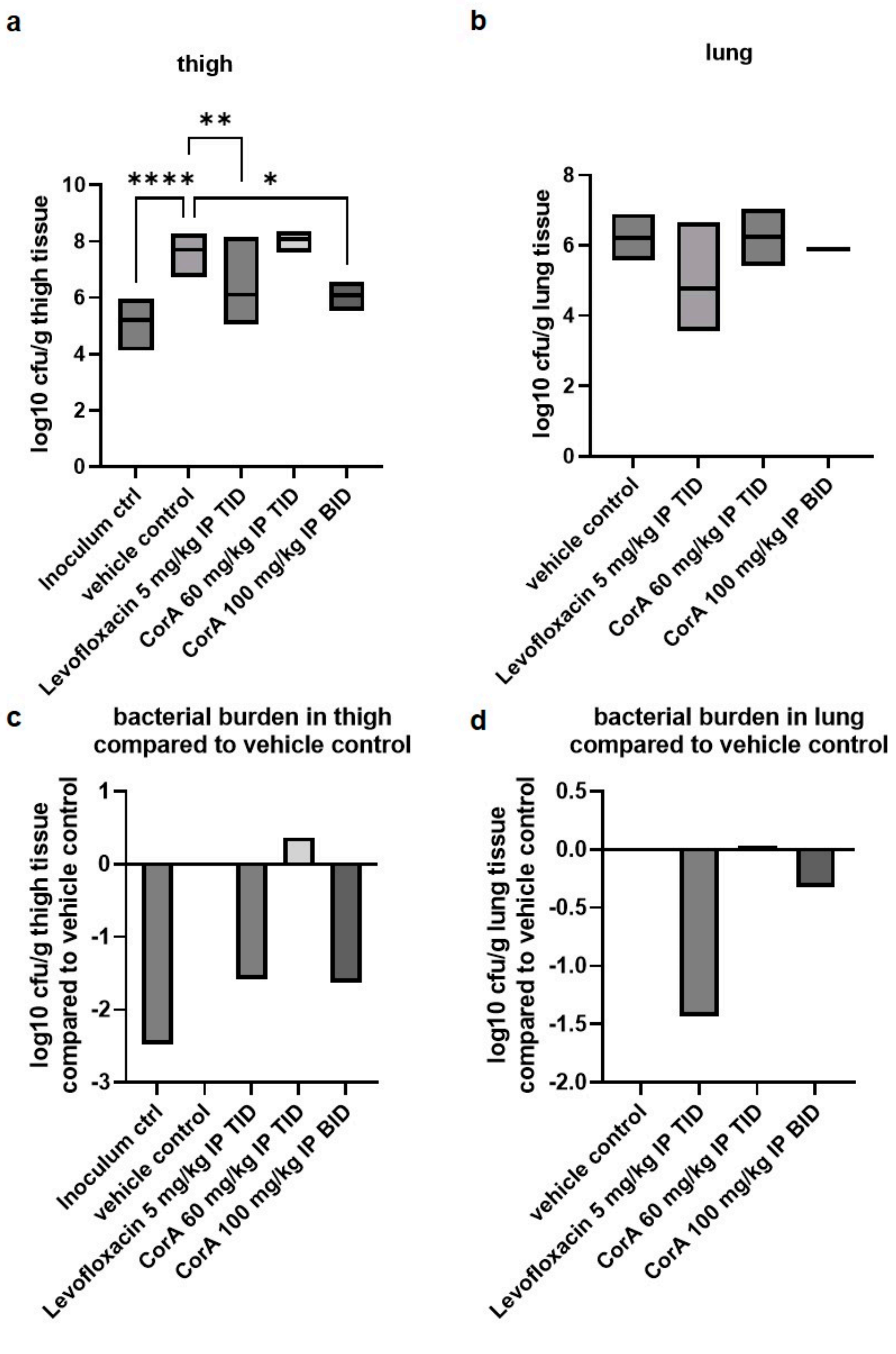

Figure S2. CorA shows efficacy in the neutropenic thigh infection model with MRSA. Bacterial burden expressed as log10 cfu/g thigh tissue is shown for inoculum control group, vehicle control group, levofloxacin 5 mg/kg IP TID, CorA 60 mg/kg IP TID and CorA 100 mg/kg IP BID. * : $p < 0.05$, **: $p < 0.01$, ****: $p < 0.0001$ using a Kruskal-Wallis test (a). Bacterial burden expressed as log10 cfu/g lung tissue is shown for vehicle control group, levofloxacin 5 mg/kg IP TID, CorA 60 mg/kg IP TID and CorA 100 mg/kg IP BID. * : $p < 0.05$ using a Kruskal-Wallis test (b). Bacterial burden reduction in thigh compared to vehicle control group is displayed for inoculum control group, vehicle control group, levofloxacin 5 mg/kg IP TID, CorA 60 mg/kg IP TID and CorA 100 mg/kg IP BID (c). Bacterial burden reduction in lung compared to vehicle control group is displayed for vehicle control group, levofloxacin 5 mg/kg IP TID, CorA 60 mg/kg IP TID and CorA 100 mg/kg IP BID (d).

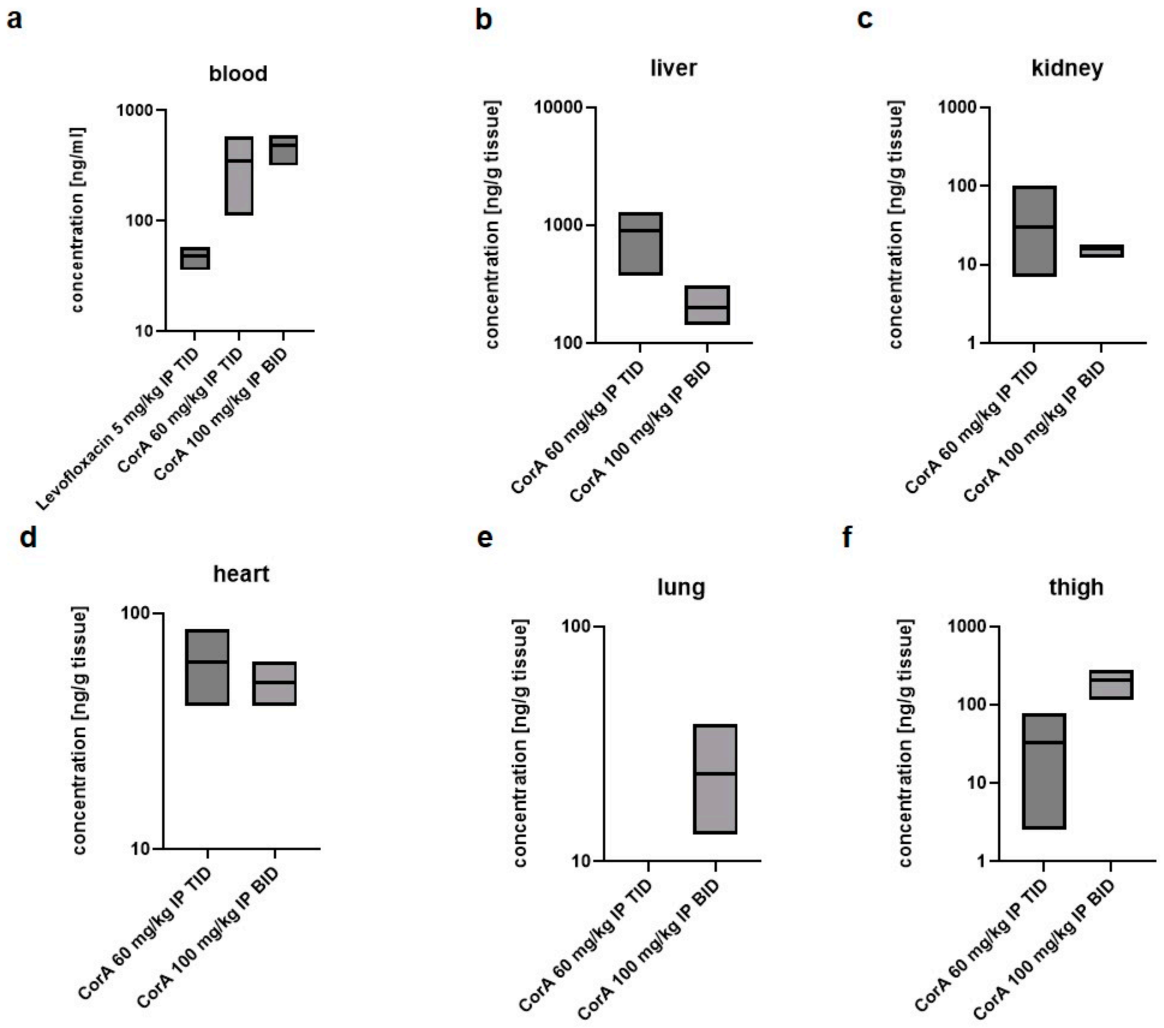

Figure S3. Bioanalytical evaluation of CorA and levofloxacin in the neutropenic thigh infection model. Compound concentrations in plasma (a), liver (b), kidney (c), heart (d), lung (e) and thigh (f) are shown for CorA 60 mg/kg IP TID, CorA 100 mg/kg IP BID and CorA 100 mg/kg SC TID. Moreover, compound concentrations in plasma (a) are displayed for levofloxacin 5 mg/kg IP TID.

Table S1. Mass transitions for quantification of CorA and levofloxacine using caffeine as internal standard.

	Q1 mass	Q3 mass	DP [volts]	CE [volts]	CXP [volts]
Caffeine	195.024	138.0	130.0	25.0	14.0
		110.0	130.0	31.0	18.0
CorA	510.147	187.1	79.0	23.0	10.0
		93.0	85.0	55.0	10.0
		121.0	80.0	21.0	14.0
Levofloxacine	361.741	318.0	60.0	25.0	16.0
		261.1	60.0	39.0	10.0

DP: declustering potential; CE: collision energy; CXP: cell exit potential.

Table S2. PK parameters in plasma after administration of 100 mg/kg PO CorA.

	100 mg/kg PO
t1/2 [h]	2.88 ± 0.6
T_{max} [h]	0.5 ± 0.4
C_{max} [μg/mL]	89.82 ± 43.8
AUC 0-t [μg/mL*h]	93.99 ± 27.1

MRT [h]	3.13 ± 0.2
Vz/F_obs [L/kg]	4.09 ± 1.3
Cl/F_obs [mL/min/kg]	16.36 ± 4.0

t1/2: plasma half-life; T_{max}: time point at which maximal concentration is reached; C_{max}: maximal concentration; AUC 0-t: Area under the curve from time point 0 until t; MRT: mean residence time; Vz/F_obs: fractionated observed volume of distribution; Cl/F_obs: observed fractionated plasma clearance; PO: perorally.
